# Supplementary material for: Polish Patients’ Needs and Opinions about the Implementation of Pharmaceutical Care in Diabetes
Source: Int J Environ Res Public Health. 2023 Jan 4;20(2):945. doi: 10.3390/ijerph20020945 (PMC9859607; doi:10.3390/ijerph20020945)
Supplement: Supplementary file 1 [file ijerph-20-00945-s001.zip › ijerph-2066876-supplementary.pdf]

UNIWERSYTET MEDYCZNY IM. KAROLA MARCINKOWSKIEGO W POZNANIU

Pracownia Farmacji Praktycznej
Katedra i Zakład Technologii Postaci Leku
ul. Grunwaldzka 6, 60-780 Poznań
tel. 61 854 66 84, e-mail: mwaszyk@ump.edu.pl

Nr ankiety…….. (Survey no…)

Szanowni Państwo! (Ladies and Gentlemen!)

Serdecznie zapraszamy do wzięcia udziału w ankiecie dotyczącej opinii pacjentów na temat edukacji farmaceuty w opiece farmaceutycznej w cukrzycy. Ankieta jest anonimowa, a jej wyniki wykorzystane zostaną wyłącznie do celów naukowych.

(We cordially invite you to take part in a survey on patients' opinions on the education of a pharmacist in pharmaceutical care in diabetes. The survey is anonymous and its results will be used for scientific purposes only.)

Właściwą odpowiedź prosimy zakreślić krzyżykiem (☒) lub wpisać w wyznaczone miejsce. Wypełnienie ankiety nie trwa dłużej niż 10 minut. Z góry dziękujemy za wzięcie udziału w badaniu.

(Please select the correct answer with a cross (☒) or write your response in the designated place. Completing the survey does not take more than 10 minutes. Thank you in advance for taking part in the study.)

**CZĘŚĆ A:** *Dotyczy ogólnych informacji, można zaznaczyć więcej niż jedną odpowiedź. (PART A: General information, more than one answer may be selected.)*		
**1. Płeć: (gender)**	**2. Wiek: (age)**	**3. Wzrost: (height)**
☐ Kobieta (Woman)<br>☐ Mężczyzna (Man)<br>☐ Inne (other)	…………………………….	…………………………………………………………..
**4.Masa ciała: (body weight)**		**5. Miejsce zamieszkania: (Place of residence)**
………………………………………………………………………		☐ Wieś (village)<br>☐ Miasto (city)

**6. Wykształcenie: (education)**				
☐ podstawowe (basic)	☐ gimnazjalne (junior high school)	☐ zawodowe (vocational)	☐ średnie (secondary)	☐ wyższe (higher)

**CZĘŚĆ B**: *(Part B)*		
**Czy ktoś u Pana/Pani w rodzinie choruje na cukrzycę? (Does anyone in your family suffer from diabetes?)**		
☐ tak (yes)	☐ nie (no)	☐ nie wiem (I don't know)

**Jak Pan/Pani ocenia swoją wiedzę na temat cukrzycy? (How do you rate your knowledge about diabetes?)**		
☐ wystarczająca (sufficient)	☐ niewystarczająca (insufficient)	☐ nie wiem (I don't know)

**Czy uważa Pan/Pani, że profilaktyka cukrzycy jest ważna? (Do you think diabetes prevention is important?)**		
☐ tak (yes)	☐ nie (no)	☐ nie wiem (I don't know)

**Jak według Pana/Pani można zapobiegać rozwojowi cukrzycy?** *Można zaznaczyć kilka odpowiedzi* **(How do you think diabetes can be prevented?** *You can mark several answers***)**	
☐ Regularna aktywność fizyczna (Regular physical activity)	☐ Kontrola poziomu cukru we krwi (Controling blood sugar levels)
☐ Spożywanie dużej ilości owoców (Eating plenty of fruit)	☐ Prawidłowe nawyki żywieniowe (Proper eating habits)
☐ Dieta oparta na produktach o niskim indeksie glikemicznym (Diet based on products with a low glycemic index)	☐ Dieta oparta na produktach o wysokim indeksie glikemicznym (Diet based on products with a high glycemic index)

**Które czynniki sprzyjają występowaniu cukrzycy typu II?** *Można zaznaczyć kilka odpowiedzi* **(Which factors contribute to the occurrence of type II diabetes***? You can mark several answers)*	
☐ Niedowaga (being underweight)	☐ Nadwaga (being overweight)
☐ Wzmożona aktywność fizyczna (Increased physical activity)	☐ Brak aktywności fizycznej (Lack of physical activity)
☐ Palenie papierosów (Smoking)	☐ Nadciśnienie tętnicze (Hypertension)

**Czy miał/a Pan/Pani badany poziom glukozy? Jeśli tak, to kiedy?** (Have you ever had your glucose level tested? If yes, then when?)	
☐ nie (no)	☐ tak, …………………..(yes)

**Czy choruje Pan/Pani na choroby przewlekłe? Jeśli tak, jakie to choroby? (Do you suffer from chronic diseases? If yes, what are the diseases?)**	
☐ nie (no)	☐ tak, (yes) ……………………………………………………………………………… ……………………

**Jakie leki Pan/Pani zażywa? (What medications are you taking?)**
………………………………………………………………………………………… ………………………………………………………………………………………… ………………………………………………………………………………………… …………………………………………………………………………………………

**Do ilu lekarzy Pan/Pani uczęszcza?** *Proszę wpisać liczbę.* **(How many doctors do you attend***? Please enter a number.)*
…………………………………………………………………………………………

**Czy chciał(a)by Pan/Pani, aby farmaceuta edukował z zakresu obsługi glukometru? Would you like the pharmacist to educate you in the use of the glucometer?**		
☐ tak (yes)	☐ nie (no)	☐ nie wiem (I don't know)

**Czy chciał(a)by Pan/Pani, aby farmaceuta edukował z zakresu podawania insuliny/ obsługi nakłuwacza? (Would you like the pharmacist to educate you on insulin administration / lancing device operation?)**		
☐ tak (yes)	☐ nie (no)	☐ nie wiem (I don't know)

**Czy chciał(a)by Pan/Pani, aby farmaceuta edukował z zakresu informacji o nowo zapisanym leku przez lekarza (usługa Nowy Lek)? (Would you like a pharmacist to provide you with information on**

**a newly prescribed drug by a doctor (New Drug service)?)**

☐ tak (yes)	☐ nie (no)	☐ nie wiem (I don't know)
**Czy chciał(a)by Pan/Pani, aby farmaceuta dokonał przeglądu lekowego? (Would you like a pharmacist to review your medication?)**		
☐ tak (yes)	☐ nie (no)	☐ nie wiem (I don't know)
**Czy chciał(a)by Pan/Pani, aby farmaceuta informował o czynnikach ryzyka cukrzycy? (Would you like your pharmacist to inform you about diabetes risk factors?)**		
☐ tak (yes)	☐ nie (no)	☐ nie wiem (I don't know)
**Czy choruje Pan/Pani na cukrzycę? (Do you suffer from diabetes?)**		
☐ tak (yes)	☐ nie (no)	☐ nie wiem (I don't know)

**CZĘŚĆ C**: *dla osób chorujących na cukrzycę* (**PART C**: *for people with diabetes)*<br><br>**Jeśli ma Pan/i cukrzycę to, którego typu? (If you have diabetes, which type?)**			
☐ Typ I (Type I)	☐ Typ II (Type II)	☐ Cukrzyca ciążowa (Gestational diabetes)	☐ Inne ………….. (Other)
**Czy poinformował(a) Pan/Pani bliskich o swojej chorobie? (Have you informed your loved ones about your illness?)**			
☐ tak (yes)	☐ nie (no)		
**W jakich okolicznościach została u Pana/Pani zdiagnozowana cukrzyca? (Under what circumstances were you diagnosed with diabetes?)**			
☐ częste oddawanie moczu (frequent urination)<br>☐ zwiększone pragnienie (increased thirst)<br>☐ nadmierne chudnięcie (excessive weight loss)	☐ osłabienie / senność (weakness/drowsiness)<br>☐ pomiar poziomu glukozy (glucose level measurement)<br>☐ badania przesiewowe (screening test)		
**Od jak dawna choruje Pan/Pani na cukrzycę? (How long have you been suffering from diabetes?)**			
…………………… lat (years)			
**Jak często bada Pan/Pani poziom glukozy we krwi? (How often do you test your blood glucose level?)**			
☐ 1 – 2 razy dziennie (1-2 times a day)<br>☐ 3 – 4 razy dziennie (3-4 times a day)<br>☐ 5 i więcej razy dziennie (5 or more times a day)	☐ Przed każdym posiłkiem (Before each meal)<br>☐ Nie sprawdzam regularnie (I don't check regularly)		
**Czy miał Pan/Pani wykonany pomiar hemoglobiny glikowanej? (Have you had your glycated hemoglobin measured?)**			
☐ tak (yes)	☐ nie (no)	☐ nie wiem (I don't know)	
**W jaki sposób jest Pan/Pani leczony/a na cukrzycę? (How are you treated for diabetes?)**			
☐ Nie leczę się farmakologicznie (I do not treat myself pharmacologically)<br>☐ 1 doustny lek przeciwcukrzycowy	☐ Wstrzyknięcia insulin (Injections of insulin)<br>☐ Osobista pompa insulinowa		

(1 Oral diabetes medication)<br>☐ Kilka doustnych leków przeciwcukrzycowych<br>(Several oral diabetes medications)	(Personal insulin pump)<br>☐ Inny: ……………………………..<br>(Other)

Ile czasu tygodniowo poświęca Pan/Pani na dodatkową aktywność fizyczną? (How much time a week do you devote to additional physical activity?)

☐ Nie uprawiam aktywności fizycznej (I do not exercise)	☐ 2 – 3 godziny (2 – 3 hours)
☐ Mniej niż 1 godzinę (Less than 1 hour)	☐ 3 – 4 godziny (3 – 4 hours)
☐ 1 – 2 godziny (1 – 2 hours)	☐ Więcej niż 4 godziny (More than 4 hours)

Jaką formę aktywności fizycznej wybiera Pan/Pani najczęściej? (What form of physical activity do you choose most often?)

☐ Spacery (walks)	☐ Fitness (fitness)
☐ Bieganie, pływanie (running, swimming)	☐ Gry zespołowe (Team games)
☐ Ćwiczenia siłowe (strength training)	☐ Jazda na rowerze (cycling)

Czy wystąpiły u Pana/Pani powikłania po cukrzycy? (Have you had complications from diabetes?)

☐ tak (yes)	☐ nie (no)	☐ nie wiem (I don’t know)

Jeśli tak, jakie to powikłania? (If yes, what are the complications?)

☐ Kwasica ketonowa (Ketoacidosis)	☐ Retinopatia (Retinopathy)
☐ Kwasica mleczanowa (Lactic Acidosis)	☐ Nefropatia (Nephropathy)
☐ Neuropatia (Neuropathy)	☐ Hiperglikemia (Hyperglycemia)
☐ Hipoglikemia (Hypoglycemia)	

Czy był/a Pan/i kiedykolwiek hospitalizowana z powodu cukrzycy? (Have you ever been hospitalized because of diabetes?)

☐ tak, z jakiego powodu? ....<br>(yes, for what reason?)	☐ nie (no)

Czy chciałby/aby Pan/i, żeby farmaceuta świadczył opiekę farmaceutyczną w diabetologii, na przykład w aptece ogólnodostępnej? (Opieka farmaceutyczna to zespół usług farmaceutycznych, które prowadzą do optymalizacji farmakoterapii oraz poprawy zależnej od zdrowia jakości życia pacjentów)
Would you like a pharmacist to provide pharmaceutical care in diabetology, for example in a community pharmacy? (Pharmaceutical care is a set of pharmaceutical services that lead to the optimization of pharmacotherapy and the improvement of patients' health-related quality of life)

☐ tak (yes)	☐ nie (no)	☐ nie wiem (I don’t know)

Według Pana/Pani objęcie opieką farmaceutyczną powinno być finansowane przez:
(In your opinion, pharmaceutical care should be financed by:)

☐ NFZ<br>(National Health Fund)	☐ prywatnie przez pacjenta<br>(privately by the patient)	☐ inne (other)

Czy chciałby/aby Pan/i korzystać z wizyt prywatnych z zakresu opieki farmaceutycznej w diabetologii? (Would you like to take advantage of private visits in the field of pharmaceutical care in diabetology?)

☐ tak (yes)	☐ nie (no)	☐ nie wiem (I don’t know)

Dziękujemy za wypełnienie ankiety!
Thank you for completing the survey!
